# Supplementary material for: Identification of the centrosomal maturation factor SSX2IP as a Wtip-binding partner by targeted proximity biotinylation
Source: PLoS One. 2021 Oct 28;16(10):e0259068. doi: 10.1371/journal.pone.0259068 (PMC8553094; doi:10.1371/journal.pone.0259068)
Supplement: S1 Fig — A, Experimental scheme. RFP-WtipN RNA (300 pg) and GFP-XSSX2IP RNA (300 pg) were coinjected into four-cell embryos. When the embryos reached stage 12.5, they were fixed, and the ectodermal tissue was imaged. B, GFP-SSX2IP localization in ectoderm cells. SSX2IP colocalizes with WtipN in cytoplasmic puncta. C, RFPWtipN localization at the junctions and cytoplasmic puncta. D-D”, Coexpression of both proteins revealed mixed cytoplasmic aggregates (arrow). Scale bar, 15 μm. Data are representative of 3 independent experiments. (PDF) [file pone.0259068.s001.pdf]

Supporting information: S1 Fig and full gel images.

S1 Fig. Colocalization of SSX2IP and WtipN in ectoderm cells.

A, Experimental scheme. RFP-WtipN RNA (300 pg) and GFP-XSSX2IP RNA (300 pg) were coinjected into four-cell embryos. When the embryos reached stage 12.5, they were fixed, and the ectodermal tissue was imaged. B, GFP-SSX2IP localization in ectoderm cells. SSX2IP colocalizes with WtipN in cytoplasmic puncta. C, RFPWtipN localization at the junctions and cytoplasmic puncta. D-D'', Coexpression of both proteins revealed mixed cytoplasmic aggregates (arrow). Scale bar, 15 μm. Data are representative of 3 independent experiments.

Fig S1

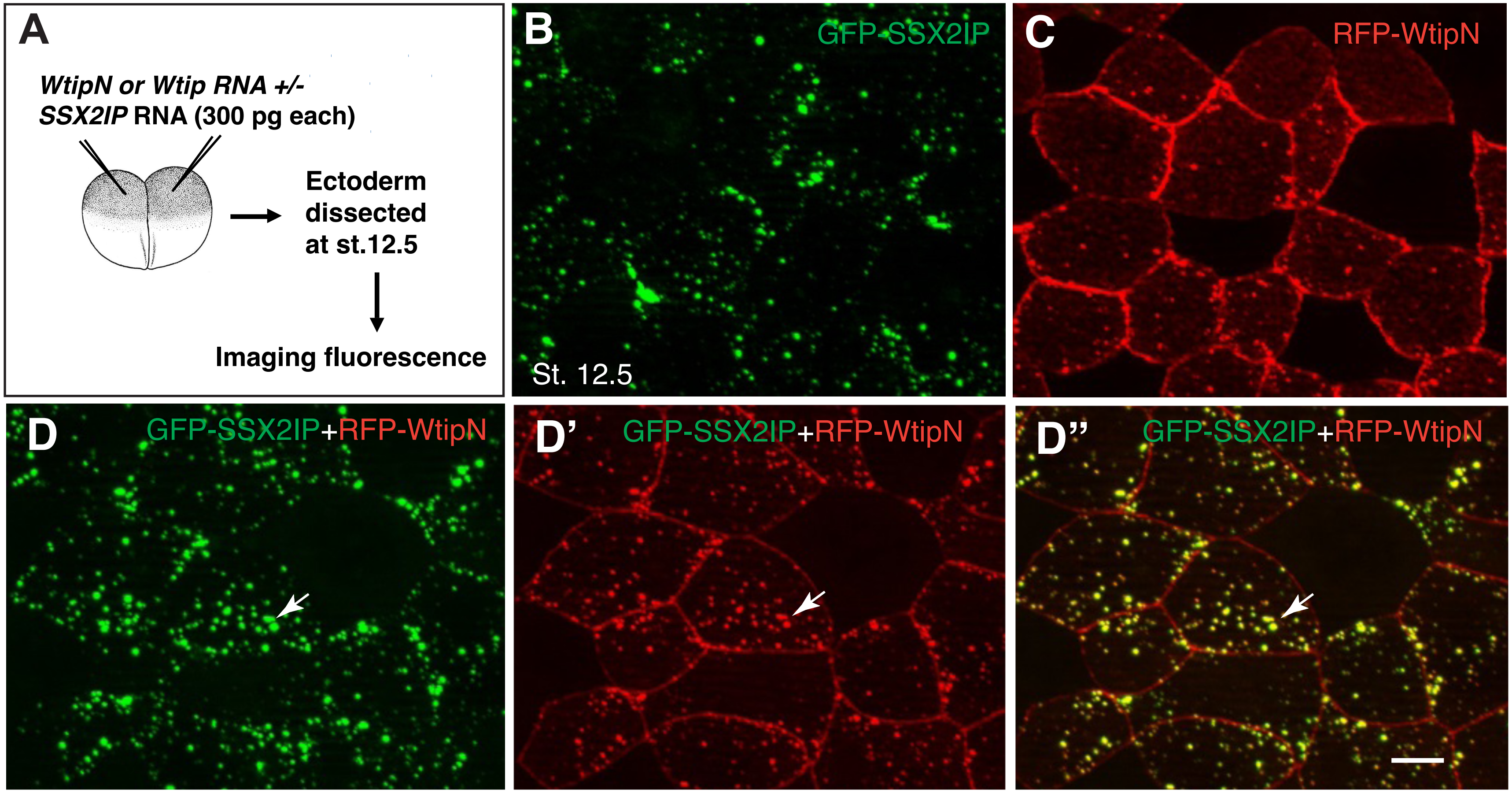
A
WtipN or Wtip RNA +/-
SSX2IP RNA (300 pg each)
Ectoderm dissected at st.12.5
Imaging fluorescence
B
GFP-SSX2IP
St. 12.5
C
RFP-WtipN
D
GFP-SSX2IP+RFP-WtipN
D'
GFP-SSX2IP+RFP-WtipN
D''
GFP-SSX2IP+RFP-WtipN
