# Supplementary figures and images for: Identification of the centrosomal maturation factor SSX2IP as a Wtip-binding partner by targeted proximity biotinylation
Source: PLoS One. 2021 Oct 28;16(10):e0259068. doi: 10.1371/journal.pone.0259068 (PMC8553094; doi:10.1371/journal.pone.0259068)

+Biotin(1.6mM)

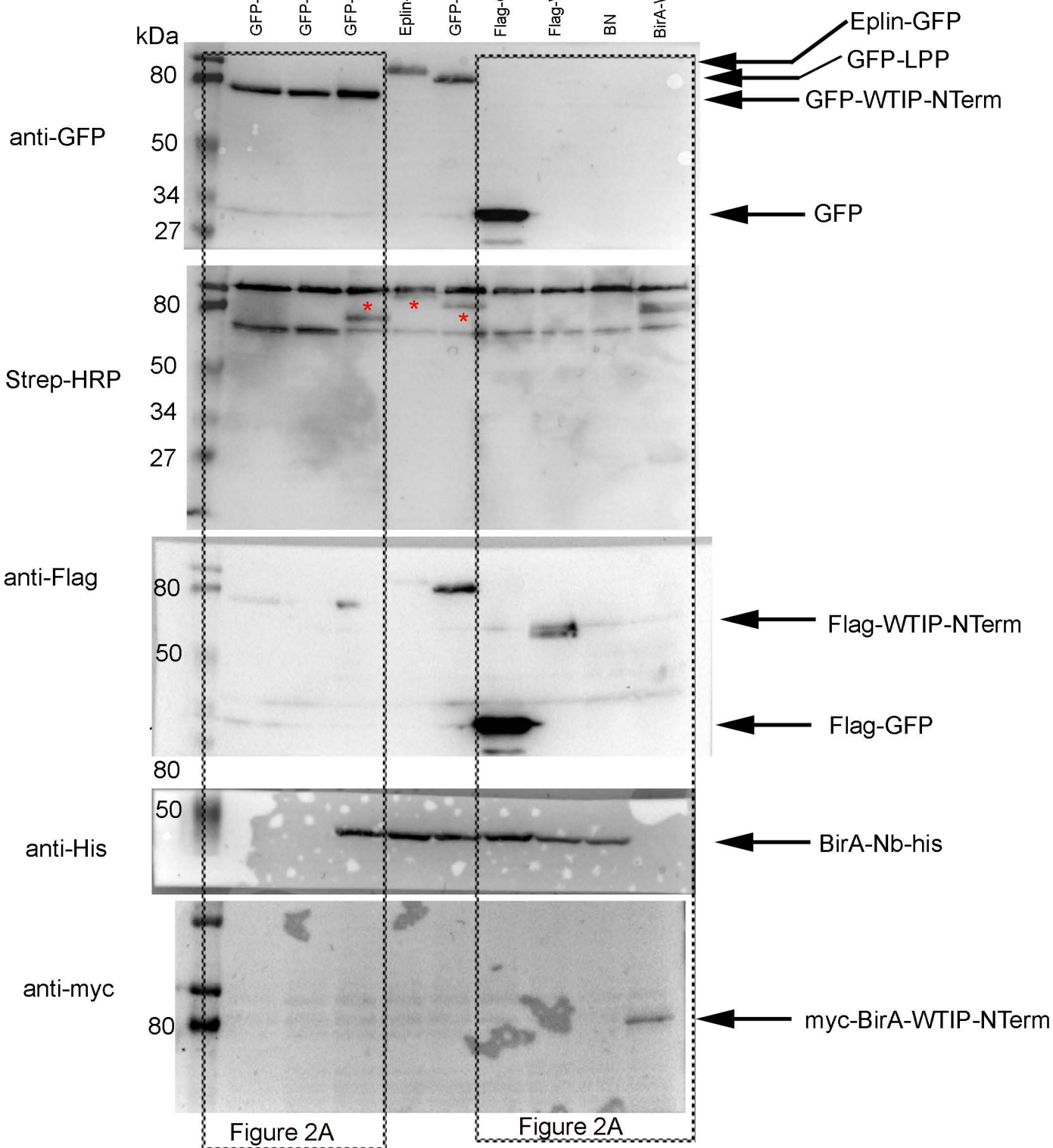

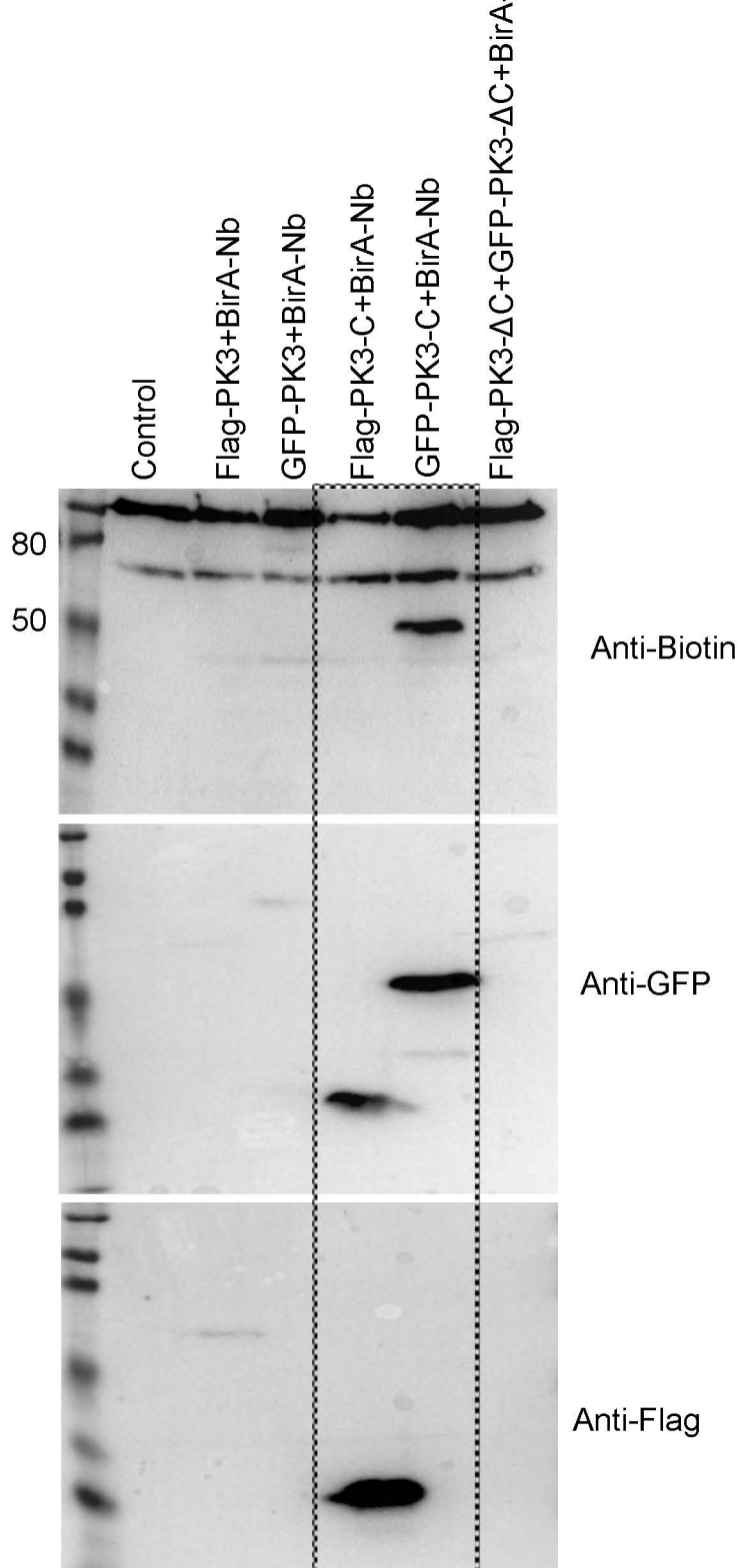

Figure 2B

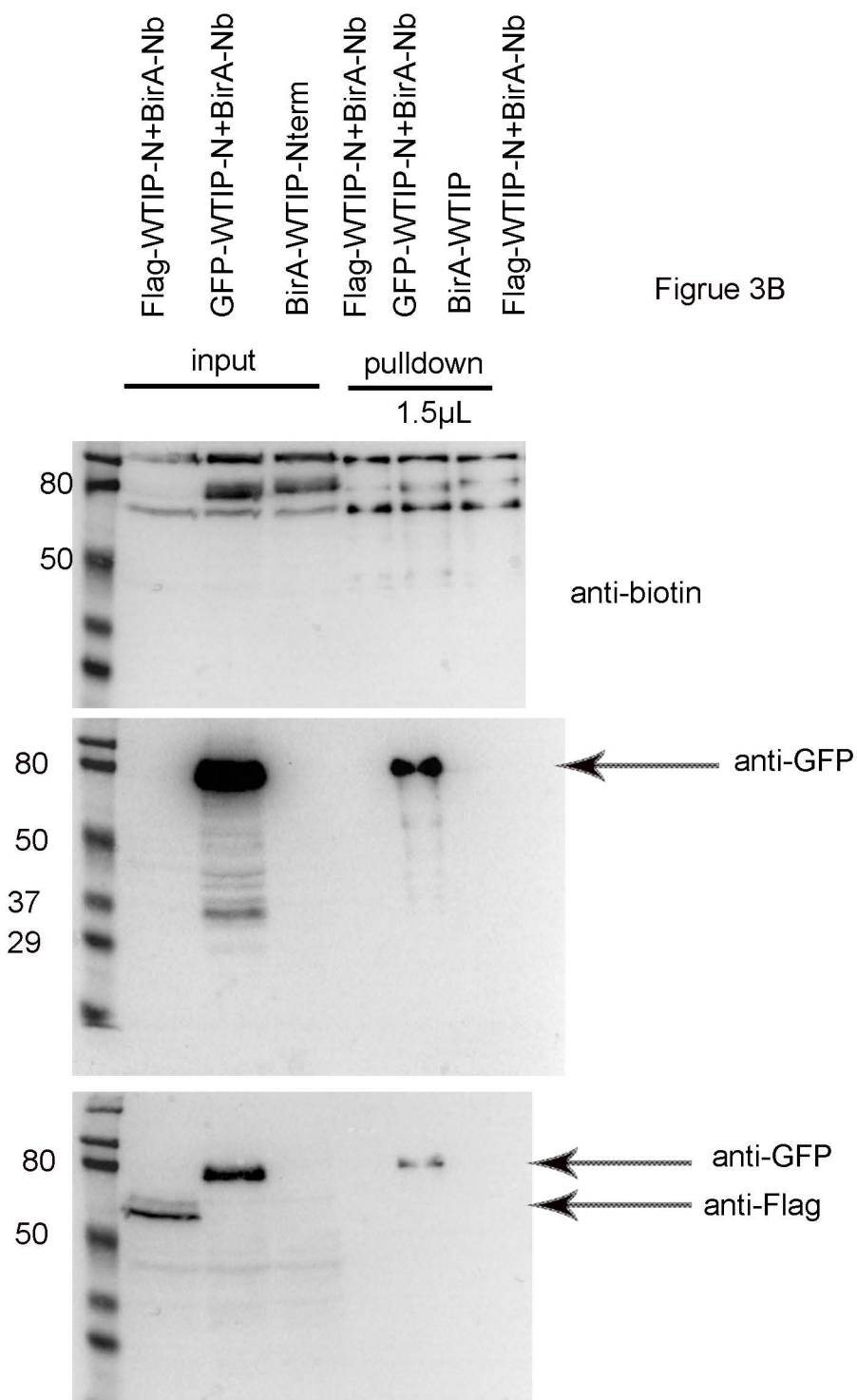

Fig. 4, Raw whole blots

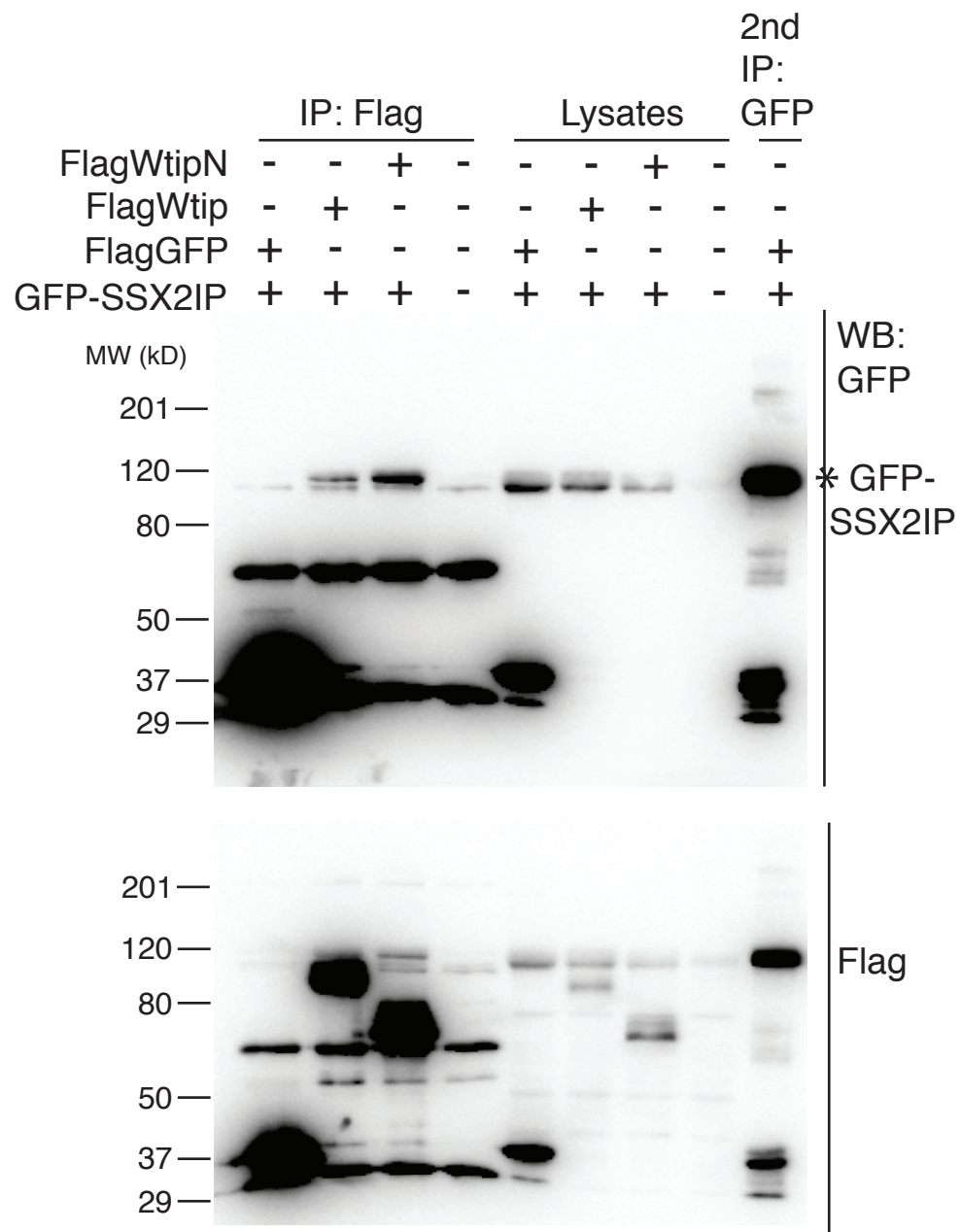

Supplement: S2 Fig — (PDF) [file pone.0259068.s002.pdf]
