# Supplementary material for: Identification of the centrosomal maturation factor SSX2IP as a Wtip-binding partner by targeted proximity biotinylation
Source: PLoS One. 2021 Oct 28;16(10):e0259068. doi: 10.1371/journal.pone.0259068 (PMC8553094; doi:10.1371/journal.pone.0259068)
Supplement: Attachment — Submitted filename: PloSOne-Reis et al.docx [file pone.0259068.s003.docx]

This is an interesting report of a new interaction between the limb domain adaptor protein Wtip (Ajuba family) implicated at the interface between the actin cytoskeleton and cell junctions, and also implicated in ciliogenesis, and SSX2IP, a protein that involved in microtubule-centrosome interactions. Specifically, the authors were looking for interactors of the N-term of Wtip, which they had previously found to be crucial for localization both at cell-cell junctions and at cilia basal bodies.

The interaction was first identified by biotinylation, pulldown and proteomics, using a protocol based on fusing an anti-GFP nanobody to the biotin ligase BirA. The authors comment on the utility of this new protocol, as it avoids fusing each bait protein with BirA, as done in standard biotinylation methods. One should note, however, that this method has its own caveats, as it failed to biotinylate a simple Flag-tagged GFP. This in my view hint at steric hindrance and/or epitope marking, which may depend on the type of GFP-fusion protein. One would wish to see this caveat included in the discussion.

As for the interaction Wtip - SSX2IP, the interaction appears robust, as validated by co-immunoprecipitation. How direct/indirect is the interaction was not investigated. A simple protein staining (silver stain?) of an SDSPAGE of the material retrieved from the biotin pulldown may be a helpful indicator of how many additional, non-biotinylated potential partners were fished.

The authors also validate the interaction by co-expression and immunofluorescence, which reveals a very convincing, extensive co-localization in cytoplasmic blobs. As expected from literature, SSX2IP is absent from the cell junctions, however one may have hoped that the Wtip-SSX2IP colocalization would happen in basal bodies.

I have here two comments:

1) Co-localization should also be performed using full length Wtip, not only its N-terminus.
2) The large number and the size of the blobs can be obviously explained by the high level of overexpression. The amount of mRNA injected (4x300pg for each of the component) is very high. It is fully justified for the pulldown as well as validation of the interaction, but using lower amounts (say 4x50, max 4x100pg) would be likely to reveal a more physiological distribution/co-localization.

Finally, two comments on the functional tests using morpholinos:

1) The standard control for specificity of MO phenotypes is a rescue by co-injection of the corresponding mRNA. This is missing here. Of course, one may not expect full rescue, as dosage + spatial targeting could be tricky, yet even partial rescue would be OK.
2) Wtip - SSX2IP co-depletion: It is here dangerous to talk about “synergy”. The results of these co-depletions could simply be explained by an additive effect. If the authors wish to argue for a functional interaction, they may want to decrease the doses of each morpholino, down to levels that show little to no phenotype when injected alone. A strong double-knock down phenotype would then be a better argument for synergistic effect.
